# *In Situ* Optical Mapping of Voltage and Calcium in the Heart

**DOI:** 10.1371/journal.pone.0042562

**Published:** 2012-08-02

**Authors:** Peter Lee, Fouad Taghavi, Ping Yan, Paul Ewart, Euan A. Ashley, Leslie M. Loew, Peter Kohl, Christian Bollensdorff, Christopher E. Woods

**Affiliations:** 1 Department of Physics, University of Oxford, Oxford, United Kingdom; 2 Division of Cardiothoracic Surgery, Papworth Hosptial, Cambridge, United Kingdom; 3 Richard D. Berlin Center for Cell Analysis and Modeling, University of Connecticut Health Center, Farmington, Connecticut, United States of America; 4 Department of Medicine, Stanford University, Stanford, California, United States of America; 5 National Heart and Lung Institute, Imperial College, London, United Kingdom; 6 Department of Computer Science, University of Oxford, Oxford, United Kingdom; University of Adelaide, Australia

## Abstract

Electroanatomic mapping the interrelation of intracardiac electrical activation with anatomic locations has become an important tool for clinical assessment of complex arrhythmias. Optical mapping of cardiac electrophysiology combines high spatiotemporal resolution of anatomy and physiological function with fast and simultaneous data acquisition. If applied to the clinical setting, this could improve both diagnostic potential and therapeutic efficacy of clinical arrhythmia interventions. The aim of this study was to explore this utility *in vivo* using a rat model. To this aim, we present a single-camera imaging and multiple light-emitting-diode illumination system that reduces economic and technical implementation hurdles to cardiac optical mapping. Combined with a red-shifted calcium dye and a new near-infrared voltage-sensitive dye, both suitable for use in blood-perfused tissue, we demonstrate the feasibility of *in vivo* multi-parametric imaging of the mammalian heart. Our approach combines recording of electrophysiologically-relevant parameters with observation of structural substrates and is adaptable, in principle, to trans-catheter percutaneous approaches.

## Introduction

Electrophysiological testing is a mainstay of clinical arrhythmia diagnosis. For simple arrhythmias, this involves a limited number of intracardiac electrogram measurements. But for complex arrhythmia, where mechanisms often are associated with underlying structural heart disease, spatially-resolved electroanatomic mapping (EAM) improves the efficacy of ablation [1]. The principle behind EAM relies on registering electrical measurements in three-dimensional space to determine the tissue location underlying the arrhythmia focus. However, EAM catalogs the spatial context of electrical signals using an external localization system and triangulates position relative to a reference patch placed externally [2], rather than by directly visualizing underlying cardiac tissue anatomy and physiology simultaneously [1].

By comparison, basic science studies of arrhythmia have tended to rely on the higher spatiotemporal resolution of optical mapping using fluorescent probes both to image anatomy directly and to measure relevant physiological parameters such as transmembrane voltage (V_m_) and intracellular calcium ([Ca^2+^]_i_) dynamics [3], [4]. Using the isolated Langendorff-perfused mammalian heart, optical mapping of these two key parameters has played a pivotal role in arrhythmia research [5], [6]. V_m_ mapping has also been used to delineate normal, peri-infarct, and scarred tissue rapidly in the *in vitro* setting [7]. Yet, the use of optical mapping *in vivo* has only been published once to our knowledge (in 1998) [8]. In our view, this is largely because it is experimentally challenging. However, a number of continued technological developments have now made the applicability of optical mapping to *in vivo* preparations more feasible, opening the door to beginning the process towards clinical development of an optical mapping tool [9]. Specifically, second-generation voltage-sensitive dyes with enhanced photostability and emission spectra in the near-infrared range have been developed, making them suitable for imaging in the presence of blood [10]. In addition, modern camera frame-rates permit simultaneous multi-color imaging using a single detector [11], in particular if combined with multi-band emission filters for optical mapping [12]. Moreover, conventional light sources can be replaced now by powerful light-emitting-diodes (LEDs) which we and others have demonstrated to offer stable illumination that can also be switched on or off with nanosecond-microsecond response times, simplifying multi-parametric imaging [13]. Based on these developments, in combination with a multi-band emission filter, we present a multi-parametric single-camera multi-LED imaging system and show its suitability for *in vivo* cardiac electrophysiology data acquisition.

## Results

**Figure 1:**
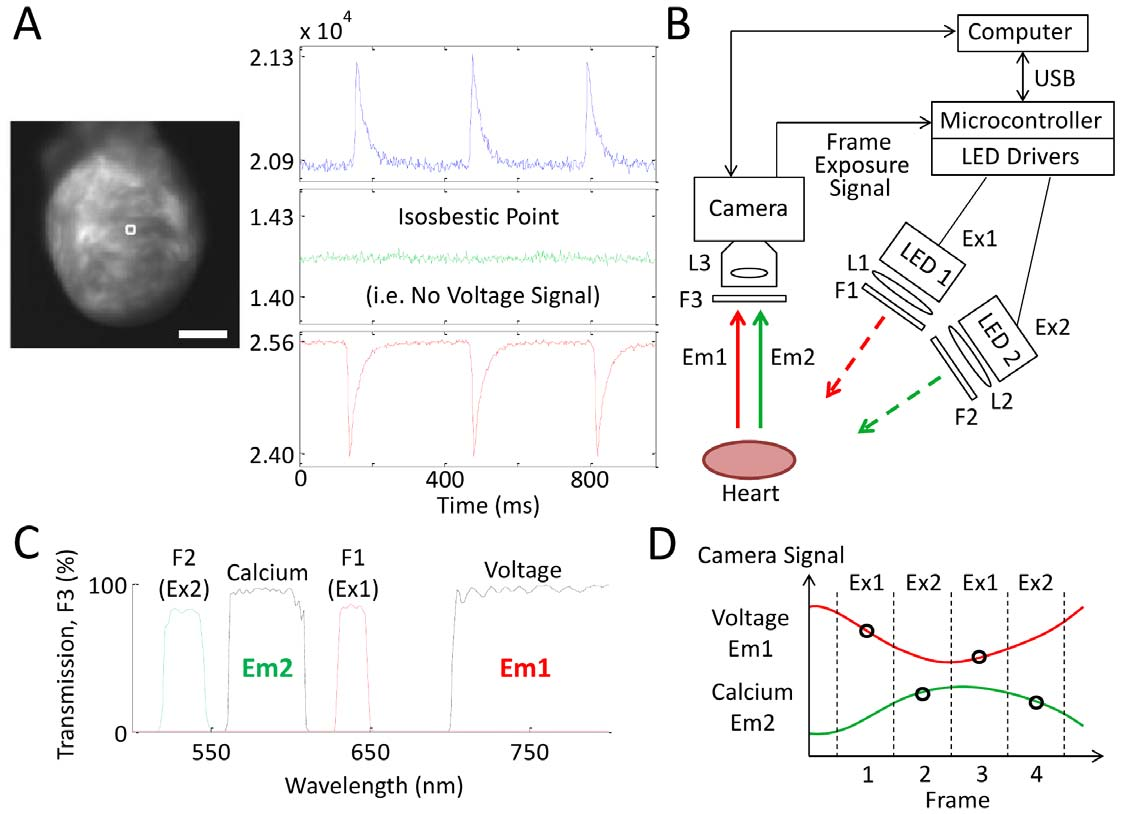
Schematic illustration of multi-parametric imaging approach. (**A**) di-4-ANBDQPQ fluorescence in a Langendorff-perfused rat heart (sinus rhythm), excited with blue (blue LED, 470±10 nm filter), green (green LED, 540±12.5 nm filter) and red (red LED, 640±10 nm filter) wavelengths. These fluorescence signals (taken from the 4×4-pixel white-square region on the left-ventricle) were collected through a custom-made multi-band emission filter (F3 in **B**, **C**). The green trace ([Ca^2+^]_i_) shows negligible emission changes when di-4-ANBDQPQ is excited at the excitation-isosbestic point. Scale bar = 5 mm. (**B**) Schematic outline of the imaging system, highlighting key components. Since only one camera is used, the system requires no challenging optical alignment. Excitation sources Ex1: red LED with a 640±10 nm filter (F1), Ex2: green LED with a 540±12.5 nm filter (F2). (**C**) Transmission spectrum of the custom multi-band emission filter that passes both V_m_ (Em1) and [Ca^2+^]_i_ (Em2) emitted fluorescence signals. F1 and F2 excitation filter spectra are shown as dashed curves. (**D**) Basic principle behind the single-camera multi-LED approach: During any frame exposure (occurring between the vertical dashed lines), the parameter being measured by the camera sensor is determined by the excitation source (either Ex1 or Ex2) that is switched on during that period. A sufficiently fast camera (compared to Em1 and Em2 signal dynamics), and interpolation between measured data points, provides simultaneous measures of multiple parameters, here V_m_ and [Ca^2+^]_i_. For these images, blebbistatin was used to eliminate the contribution of motion to the signals.

We chose di-4-ANBDQPQ [10] to report V_m_, and rhod-2(AM) to report [Ca^2+^]_i_ changes, as both dyes had previously been used successfully in diluted-blood perfused Langendorff hearts [14]. To avoid cross-talk between dye emissions, we made use of the ratiometric properties of di-4-ANBDQPQ [10], [12]. As shown in Figure 1A, excitation of this dye with either blue or red wavelengths produces action potential (AP) fluorescence signals that increase or decrease with changes in V_m_, respectively (although not used in this study, ratiometric V_m_ imaging can be used to reduce motion artifacts). During exposure to green light (used for excitation of rhod-2(AM)) there is no change in voltage-related emission, as this occurs at the excitation-isosbestic point for di-4-ANBDQPQ. This approach allows simultaneous V_m_ and [Ca^2+^]_i_ imaging (Figure 1) without emitted signal cross-talk.

A schematic illustration of the overall single-camera imaging/multiple-LED excitation system is shown in Figure 1B. The multi-band emission filter used (F3 in Figure 1B) was custom-fabricated by Chroma Technology (Bellows Falls, VT, USA) to provide transmission bands for fluorescence emitted from both di-4-ANBDQPQ (Em1) and rhod-2(AM) (Em2; Figure 1C). Figure 1D illustrates the principle behind our approach: During one camera frame-exposure, excitation light 1 (Ex1) is turned on, producing V_m_ signal emission collected through the Em1-band; during the next frame exposure, excitation light source 2 (Ex2) is turned on, producing [Ca^2+^]_i_ signal emission collected through the Em2-band, and so on. Since the camera frame rate (here >500 frames-per-second; kHz frame rates can be achieved with pixel-binning) is faster than physiological signal dynamics, with interpolation, one can record electrophysiologically-relevant dynamic changes in both V_m_ and [Ca^2+^]_i_ (such as the action potential [AP] and the calcium transient [CaT]), and assess AP and CaT propagation.

**Figure 2:**
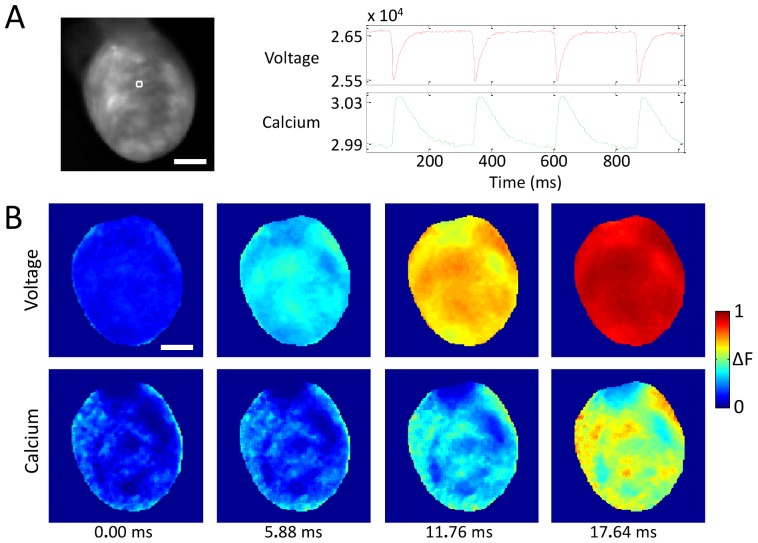
Simultaneous imaging of V_m_ and [Ca^2+^]_i_ in a Langendorff-mode saline-perfused rat heart. (**A**) V_m_ (red) and [Ca^2+^]_i_ (green) fluorescence signals (camera signals on a 16-bit scale) taken from the 4×4-pixel white-square region on the left ventricle. (**B**) Normalized fluorescence intensity maps (colorbar shown) at progressive time points during sinus rhythm. The delay of the CaT relative to the AP peak (∼17 ms from transients in part **A**) is clearly visible. Scale bar = 5 mm. For these images, blebbistatin was used to eliminate the contribution of motion to the signals.

Figure 2 shows results obtained using the imaging system in proof-of-principle work on saline-perfused rat hearts in Langendorff-mode. In Figure 2A, AP and CaT fluorescence signals from one location on the left ventricle are plotted, while Figure 2B shows a time series of normalized V_m_ and [Ca^2+^]_i_ fluorescence intensity maps, highlighting the well-known delay (here ∼17 ms) of the CaT peak relative to AP upstroke (see Movie S1). Figure S1 shows AP and CaT fluorescence signals from four other points on the same heart, demonstrating signal quality across the whole field of view. Unless otherwise stated for these images and what follows, blebbistatin was used to reduce the contribution of motion to the fluorescence signals.

**Figure 3:**
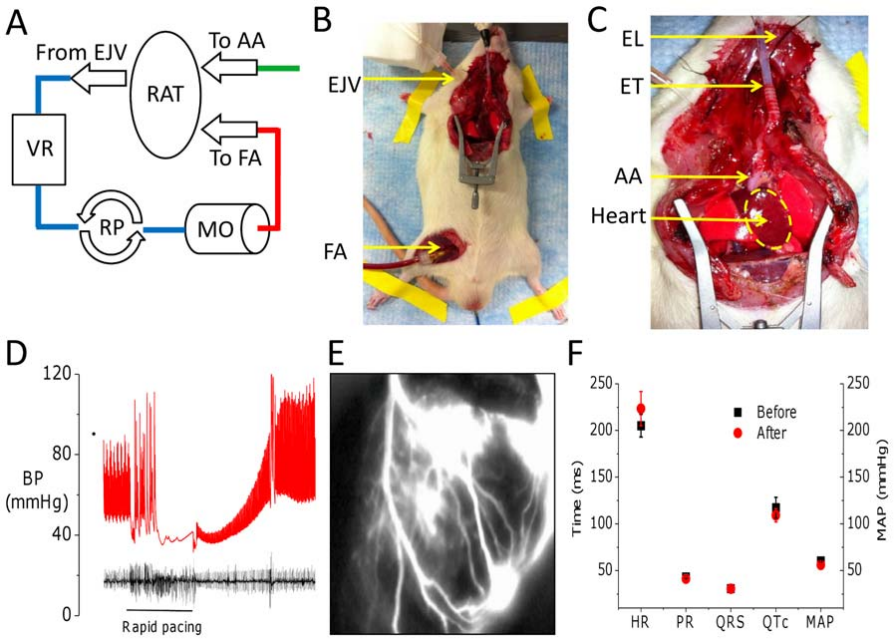
*In vivo* rat whole-heart preparation. (**A**) A schematic of the rat cardiopulmonary bypass (CPB) circuit based on a standard pump circuit (EJV: right external jugular vein catheter, VR: venous reservoir, RP: roller pump, MO: miniature membrane oxygenator, FA: right femoral artery catheter, AA: ascending aorta catheter via right carotid artery). In CPB mode, blood is pumped from the EJV through the MO to the FA, epicted by arrows in the figure. Dye is injected through the AA catheter. (**B**) Whole-animal view of the preparation. (**C**) Zoomed-in view of the open chest, with the heart in clear view (EL: esophageal ECG lead, ET: endotracheal tube). (**D**) An example of RVP at 12 Hz to drop blood pressure (BP; red trace), after which dye was injected via the aortic root. The BP recovered soon after cessation of pacing. Immediately after loading as shown here, the BP increased reflecting the Frank-Starling effect of prolonged loading. The accompanying ECG (black trace) is also shown. (**E**) Zoomed-in view of the heart immediately after rhod-2(AM) injection demonstrating a fluorescent coronary angiogram. (**F**) Pooled data on heart rate (HR in ms), mean arterial BP (MAP in mmHg—right axis), as well as far field EKG parameters of PR, QRS, and QTc intervals (in ms) for immediately before (black) and at 5 minutes after loading with voltage dye (red).

**Figure 4:**
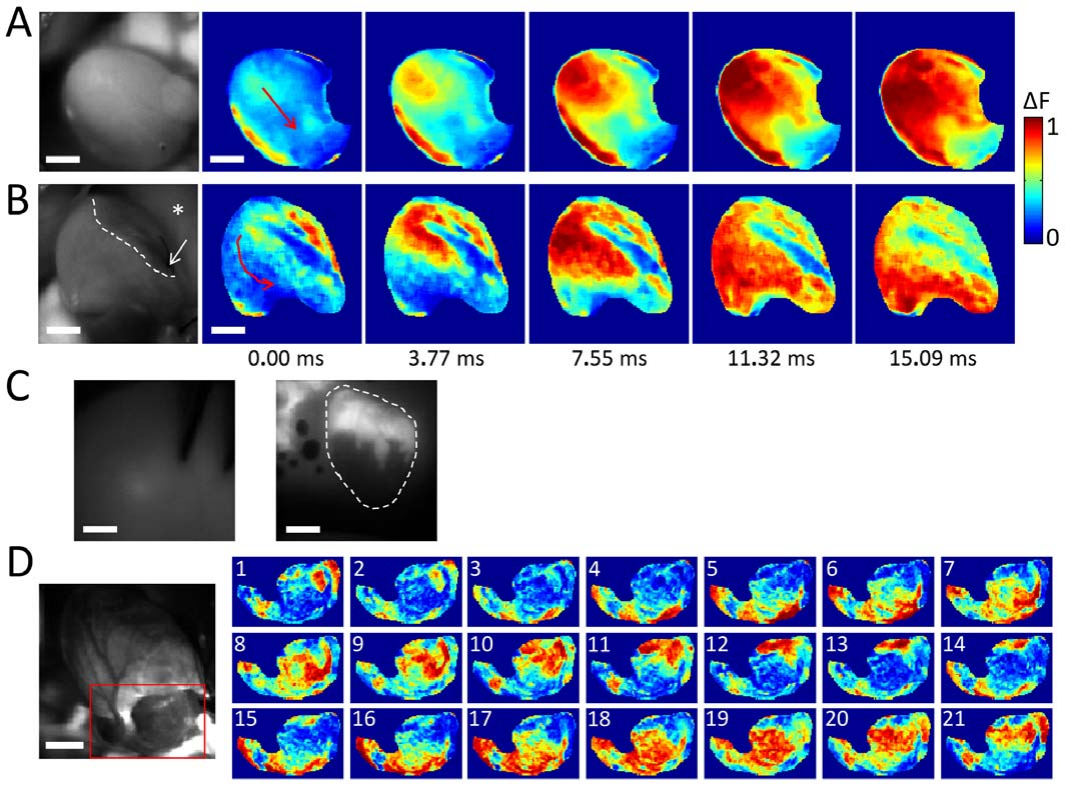
*In vivo* imaging of V_m_ and [Ca^2+^]_i_ dynamics in rat ventricles/atrium, during sinus rhythm and in atrial fibrillation. (**A**) Normalized V_m_ fluorescence intensity maps (colorbar shown) at progressive time points of the cardiac cycle with the heart in sinus rhythm (red arrow indicates electrical wave propagation direction). (**B**) Normalized V_m_ fluorescence intensity maps at sequential time points of the heart in sinus rhythm, 1 hour after suture-ligation of the proximal LAD (to mimic myocardial infarction; red arrow indicates electrical wave propagation direction). In the left-most (raw grayscale) panel, the infarcted tissue can be recognized as the dark region (one side demarcated by dotted white line) in the top-right quadrant of the image; in the normalized fluorescence maps it is recognized as the persistently blue region in the same area. Suture location is indicated with a white arrow. The asterisk marks lung tissue. (**C**) Endocardial view of same infracted heart as in (**B**), after necropsy, submerged in blood (left) and with a coverslip gently placed on the tissue (right). The bright areas correspond to non-infarcted tissue, while the dark areas correspond to infarcted tissue where the dye is absent. The dashed white line demarcates heart tissue border. Note the air bubbles on the left side of the panel at the coverslip-tissue interface. (**D**) Normalized [Ca^2+^]_i_ fluorescence intensity maps at sequential camera frames in part of the left and right atria during atrial fibrillation, induced by global ventilatory hypoxia. Scale bar = 5 mm. For **A** and **B**, blebbistatin was used to reduce, but not eliminate, the contribution of motion to the signals, while for **C**, no blebbistatin was used. Tissue shown in **B** and **C** correspond to a different heart than that shown in **A**.

Next, we applied our imaging system to the rat heart *in vivo* using di-4-ANBDQPQ and rhod-2(AM) in isolation. Figure 3 shows the *in vivo* preparation used (details can be found in the **Materials and Methods** section). In Figure 3F, we show summed results of the heart rate (in ms), PR, QRS, and QTc intervals for far field EKG recordings immediately before and 5 minutes after loading of voltage dye. We found no appreciable differences in these EKG traces (n = 3). In addition, mean arterial pressure (MAP) is shown again immediately before and 5 minutes after loading with voltage dye. There was no appreciable effect on MAP with voltage dye loading. As noted in the **Materials and Methods** section, to reduce motion contamination in both the V_m_ and [Ca^2+^]_i_ fluorescence, blebbistatin was added during imaging. With the addition of blebbistatin we used cardiopulmonary bypass (CPB) circuit to maintain blood pressure as described in the **Materials and Methods** section. Figure 4A shows normalized V_m_ fluorescence intensity maps at progressive time points in a heart *in vivo* (see Movie S2). Figure 4B shows normalized V_m_ fluorescence intensity maps at progressive time points 1 hour after LAD suture ligation to induce local myocardial infarction (to be differentiated from scar) (see Movie S3). In the left panel, a raw grayscale fluorescence image of V_m_ signal emission during diastole shows that the infarcted tissue is darker than surrounding normal myocardium (upper right quadrant), as the dye cannot reach the infarct. In the normalized V_m_ fluorescence intensity maps, AP propagation proceeds through viable tissue (best appreciated in Movie S3). In Figure 4C, we show a necropsy specimen of the endocardial surface of the same heart, highlighting V_m_ emission differences at the normal (bright) and infarcted (dark) tissue. The left panel in Figure 4C shows the specimen submersed beneath ∼1 cm of blood, while on the right, a coverslip has been placed on the tissue (bubbles highlight the coverslip-tissue interface). This gross demonstration of normal versus infarct tissue illustrates the ability of the dye to differentiate between the two tissue types rapidly. Clearly, collateralization of infarcted tissue by remodeling, and identification of scar from viable tissue will be critical to investigate, as has been done using voltage dyes *in vitro* [7]. But, this was not possible in this small animal model because, while the LAD infarct did indeed lead to scar formation (as confirmed in subsequent cardiac MRI studies conducted in hearts; data not shown), we did not see collateralization in our infarct model. In Figure 4D, we show an example of calcium fluorescence measurements *in vivo*. Here, normalized [Ca^2+^]_i_ fluorescence intensity maps are shown for the epicardial surface containing the atria. The activity shown is during atrial fibrillation. Because the electrical activation frequency of atrial fibrillation is exceedingly high and (∼8–9 Hz in the rat) and disorganized, atrial contraction is uncoordinated. While this is detrimental clinically, imaging is improved as movement is minimized (no blebbistatin needed; see Movie S4).

**Figure 5:**
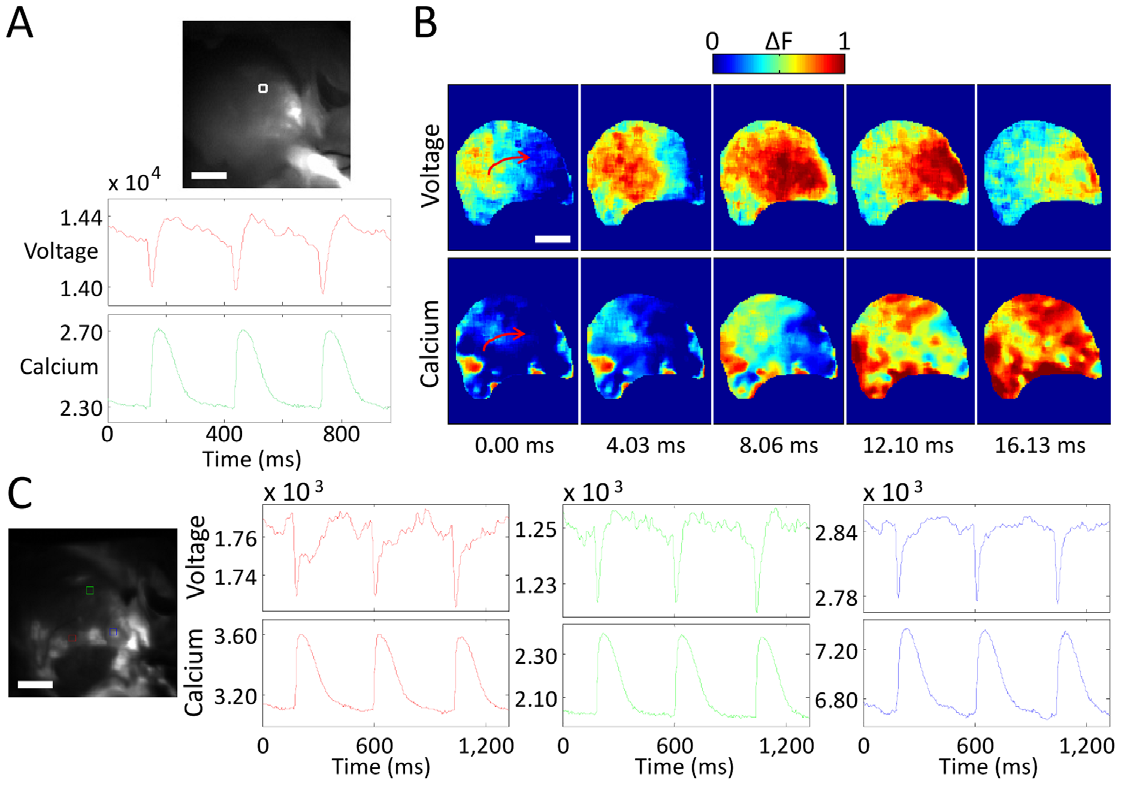
*In vivo* imaging of V_m_ and [Ca^2+^]_i_ dynamics in rat ventricles during sinus rhythm. (**A**) AP (red) and CaT (green) fluorescence signals taken from the 4×4-pixel white-square region highlighted in the top panel (left ventricle). (**B**) Normalized fluorescence intensity maps (colorbar shown) for V_m_ and [Ca^2+^]_i_ during sinus rhythm from the same heart; note the previously mentioned delay between V_m_ and [Ca^2+^]_i_ peaks (red arrow indicates electrical wave propagation direction). (**C**) AP (top traces) and CaT (bottom traces) fluorescence signals taken from three 4×4-pixel square regions from the left ventricle of another heart, showing varying motion artifact effects. Scale bar = 5 mm. For these images, blebbistatin was used to reduce, but not eliminate, the contribution of motion to the signals. For **B**, non-uniform dye-loading and motion artifact are the cause of signal quality heterogeneity.

In the final set of experiments, we dual-loaded the *in vivo* heart with both rhod-2(AM) and di-4-ANBDQPQ. Figure 5A shows V_m_ (red) and [Ca^2+^]_i_ (green) fluorescence signals from a point on the left ventricle, demonstrating AP and CaT measurements (some motion artifacts present in signals). Figure 5B shows normalized V_m_ and [Ca^2+^]_i_ fluorescence intensity maps at progressive time points from the same heart (see Movie S5). Figure 5C shows V_m_ (top traces) and [Ca^2+^]_i_ (bottom traces) fluorescence signals from three points on the left ventricle of another heart, demonstrating varying motion artifact effects when contraction is not completely eliminated. In contrast to the loading of di-4-ANBDQPQ, during which hemodynamic effects were not seen, rhod-2(AM) application by direct root injection required CPB support, as the [Ca^2+^]_i_ dye could lead to asystolic arrest during initial injection, with subsequent beating recoverable with circulatory support (which was always needed for calcium dye experiments). Dye loading protocols *in vivo* need to be optimized. Our data, however, shows that it is possible in principal to conduct combined V_m_ and [Ca^2+^]_i_ imaging in the blood-perfused mammalian heart *in vivo*.

## Discussion

Here, we introduce a multi-parametric imaging approach, building on a near-infrared voltage dye and red-shifted calcium dye, which relies on a multi-band filter to study two key parameters responsible for arrhythmia *in vivo* using a single camera. The technique, also suitable for Langendorff-perfused hearts, is scalable and less expensive than traditional optical mapping methods. In contrast to the single previous report on *in vivo* optical mapping [8], our system is adaptable, in principle, to trans-catheter endoscopic methods, such as used to visualize the endocardium in humans [15], [16], [17], [18], opening the door to clinical development as an arrhythmia mapping tool.

Because EAM suffers from suboptimal spatiotemporal resolution and can be very time consuming [19], [20], alternative invasive modalities have been developed to circumvent some of these limitations. As an example, direct endocardial visualization through optical catheters can simplify the procedure of ablation by relying on visualized anatomy through a saline-filled balloon [21], [22]. However, visualization catheters do not report complex electrical activity by comparison to EAM. With its high spatiotemporal resolution, optical mapping may meet this need by allowing for complex electrophysiology and direct anatomy to be mapped simultaneously through a minimally invasive approach. In addition, because optical catheters can visualize ablation lesions [16], in combination with optical mapping, it may be possible to visualize the interplay of ablation lesions and electrical block. Optical mapping also allows [Ca^2+^]_i_ measurements, a parameter not accessible to traditional EAM. Given the interplay of V_m_ and [Ca^2+^]_i_ in arrhythmogenesis [6], clinically mapping this parameter could be fruitful.Finally, optical mapping can be conducted beat by beat, allowing for mapping of transient arrhythmias or infrequent ectopic events.

Our study has several limitations. First and foremost, we have yet to couple our imaging system to a direct visualization catheter. Second, in our experiments, excitation-contraction un-couplers were needed to reduce motion artifact from fluorescence transients, which is undesirable. However, motion correction algorithms [23] and ratiometric imaging of fluorescence transients [24] have been used extensively to deal with motion artifacts in signals. In the isolated heart preparation, mechanical restriction or ratiometric imaging with post-acquisition motion tracking has been shown to significantly reduce motion artifacts, but imaging a strongly contracting whole-heart is still an unsolved problem [9]. Indeed, an even simpler approach has been used with direct visualization catheters where local mechanical immobilization by the endocardial balloon successfully limits motion artifacts [17], even as applied to high resolution laser based ablation in patients [25]. Third, while we found no evidence of short-term detrimental effects of di-4-ANBDQPQ, long term toxicity studies need to be done. However, the recent track record in voltage-sensitive dye development has been impressive, making us optimistic overall. Fourth, the calcium arrest seen during *in vivo* loading is thought to be caused by the calcium-buffering properties of the dye on either intra- or extracellular calcium levels. Further exploration of lower-affinity calcium dyes with red-shifted emissions may prove fruitful. In spite of these significant limitations discussed above, this proof-of-principle investigation confirms that ‘optical EAM’ is possible *in vivo* and argues for continued development.

## Materials and Methods

### Ethics statement

Studies were approved by the Stanford University Administrative Panel on Laboratory Animal Care and conform to the Guide for the Care and Use of Laboratory Animals published by the National Institutes of Health.

### Imaging system

Our system was constructed from readily-available optical filters, lenses, LEDs, electronics, and a single high-speed, electron-multiplied charge-coupled-device (EMCCD) camera (Photometrics, Tucson, AZ, USA). We measured V_m_ using di-4-ANBDQPQ (Berlin Center for Cell Analysis and Modeling, University of Connecticut, USA) and [Ca^2+^]_i_ using rhod-2(AM) (Molecular Probes, Eugene, OR, USA). Di-4-ANBDQPQ was excited with Ex1: LED CBT-90-R (peak power output 32 W; peak wavelength 628 nm; Luminus Devices, Billerica, MA, USA) using excitation filter D640/20× (Chroma Technology, Bellows Falls, VT, USA). Rhod-2(AM) was excited with Ex2: LED CBT-90-G (peak power output 58 W; peak wavelength 524 nm; Luminus Devices) with excitation filter D540/25× (Chroma Technology). Light from the LEDs was collimated with plano-convex lenses (LA1951; Thorlabs, Newton, NJ, USA). Fluorescence emission from dye-loaded hearts was passed through custom-made multi-band emission filter F3, available from Chroma (ET585/50-800/200 m; Chroma Technology), with high-percentage transmission spectra for both emission bands (∼560–610 nm and ≥700 nm), and collected with a fast camera-suitable lens (f/# 0.95; DO-2595; Navitar). Fluorescence images were taken with a high-speed EMCCD camera (Cascade® 128+ or Evolve™ 128, Photometrics) running at 510 (Cascade) or 530 (Evolve) frames-per-second (both at 128×128 pixels).

A microcontroller-based interface was implemented to synchronize excitation light switching with EMCCD camera frame exposure periods (in our experiments, the camera ran off its internal clock; i.e. the camera was master and the LEDs were slaves). LEDs were controlled with a custom-built high-power LED driver circuit that enables illumination power to be tuned, and light output to be switched in the kHz range. An eight-processor microcontroller (Propeller chip; Parallax, Rocklin, CA, USA) was used to control and coordinate all major components of the set-up. Control software for time-critical tasks was written in the microcontroller's assembly language, to ensure a time resolution of 50 ns. Communication with a standard desktop computer was implemented via a USB interface module (UM245R; Future Technology Devices International, Glasgow, UK). Custom software, written in MATLAB (The MathWorks, Natick, MA) was used to design experiments, to communicate with the microcontroller, and to perform optical mapping image processing. All electronic components used to build the microcontroller-based interface were acquired from major electronic components distributors (e.g. Digi-Key, Thief River Falls, MN, USA). Circuit schematics can be made available upon request.

### Isolated Langendorff-perfused rat heart

Hearts from female Wistar rats (n = 3) weighing 250–350 g were isolated after cervical dislocation in accordance with Schedule 1 of the UK Home Office Animals (Scientific Procedures) Act of 1986, and swiftly connected to a Langendorff setup. Hearts were perfused at a constant rate of 5 mL/min with Tyrode's solution (containing, in mmol/L: NaCl 140, CaCl_2_ 1.8, KCl 5.4, MgCl_2_ 1, Glucose 11, HEPES 5; bubbled with oxygen; pH 7.4). All chemicals were obtained from Sigma-Aldrich (Dorset, UK) if not otherwise stated.

Fluorescent dyes were sequentially injected into the aortic cannula for coronary perfusion. Hearts were first stained by re-circulating perfusion with 50 mL of 10 µmol/L rhod-2(AM) for 30 min. The myocardium was then stained by delivering, without recirculation, a 20 µL bolus of 27.3 mmol/L (in pure ethanol) di-4-ANBDQPQ, applied over 5 min (i.e. diluted in 25 mL perfusate). [10] To improve di-4-ANBDQPQ loading, Pluronic F-127 (Molecular Probes) was added to the bolus at a final concentration of 0.2–0.5%.

Excitation-contraction coupling (ECC) was blocked for some of the final imaging studies with blebbistatin (Sigma-Aldrich), using a concentration of 10 µmol/L [26]. All experiments were conducted at 36±1°C.

### *In vivo* rat animal model

Male Sprague-Dawley rats (250–350 g) were anesthetized with 1–2% isoflurane in oxygen. Rats were maintained at 37°C and depth of anesthesia was monitored throughout according to standard protocols (APLAC, Stanford University). Tracheotomy was established with a 14-gauge cannula and the animal was ventilated at a pressure support of 15 mmHg at 60 breaths per minute (Kent Scientific, San Diego, CA, USA). A standard sternotomy was then performed. The right femoral artery (FA), external jugular vein (EJV), and ascending aorta (AA), via the carotid artery (CA), were cannulated with polyethylene tubing. Heparin (1,000 units/kg, Sigma-Aldrich) was administered intravenously (Figure 3). A 1.4 Fr conductance catheter (Millar Instruments, Houston, Texas, USA) was introduced via the esophagus to provide an ECG lead. Blood pressure was monitored with a pressure transducer (Hugo Sachs Electrical, March-Hugstetten, Germany) attached to the side-port of the FA cannula. For experiments with ventricular pacing, a pacing stimulator (Millar Instruments) was placed on the left ventricular epicardial apex. Dyes were loaded as specified in the text via the CA catheter. For select experiments as specified in the text, a cardiopulmonary bypass (CPB) circuit was used to maintain a mean coronary perfusion pressure of 60 mmHg, on average (Figure 3A) [27]. The CPB circuit involved a micro-peristaltic pump (Digital Reglo Pump Model 1329, Ismatec Ltd., Wertheim-Mondfeld, Germany), in line with the tubing and a miniature membrane oxygenator (Living Systems Inc St. Albans, Vermont, USA), and in parallel to a venous reservoir, which was utilized to avoid air leaks. The circuit was primed with 5 mL of heparinized saline (20 units/mL) to remove all air. The CPB circuit was attached in series to the EJV and FA, and run in a retrograde manner when used.

Figures 3B and 3C show photographs of the imaging camera view of the preparation. Traditional coronary catheterization has already been employed to load voltage dyes in larger animals without complications [8], but was impractical in the rat. Therefore, dyes were injected into the ascending aorta via the carotid artery and towards the coronary circulation. To accomplish this, blood pressure had to be reduced to allow sufficient coronary perfusion with the dyes. As shown in Figure 3D and 3E, blood pressure was reduced through combination of rapid ventricular pacing (RVP, 12 Hz) to impair ventricular diastolic filling, thereby reducing stroke volume sufficiently to decrease arterial blood pressure, similarly to what done clinically during stent-valve implantation [28]. Once blood pressure was reduced sufficiently to decrease resistance to injection (Figure 3D), the aorta was temporarily cross-clamped, and dye was injected through the carotid artery catheter, mimicking the clinical aortic root injections of contrast agents [28]. Figure 3E shows a still image during dye loading (rapidly thereafter the dye leaves the vascular space because of its amphiphilic properties. We found that RVP and cross-clamp periods not exceeding 10 seconds were sufficient to permit dye loading through the coronary circulation. Blebbistatin was added to the circulation during final imaging to reduce wall motion [26]. When blebbistatin was used, cardiopulmonary bypass was used to preserve coronary perfusion.

Single V_m_ mapping experiments (n = 3) were possible within ∼5 min of dye loading, while dual V_m_/[Ca^2+^]_i_ mapping (n = 3) required ∼30 min before imaging (prolonged waiting time necessary for rhod-2(AM) de-esterification inside the cells; di-4-ANBDQPQ was loaded after rhod-2(AM)). For the coronary infarct model, the proximal left anterior descending coronary artery (LAD) was ligated with a 4-0 suture. 1 hour was allowed to pass to mimic acute myocardial infarction prior to imaging, after which dye was injected as described above (in the presence of the tied-off LAD ligature).

### Image processing

Custom MATLAB software was used to perform optical map image processing. Signals were filtered (in time) with local regression using weighted linear least squares and a 1^st^ degree polynomial model (MATLAB's built-in *smooth* function) and images were filtered using 2D median filtering (MATLAB's built-in *medfilt2* function).

## Supporting Information

Figure S1 **More Optical transients of** **Figure 1** **.**
(DOC)

Movie S1 **Optical mapping video of** **Figure 2B** **.**
(WMV)

Movie S2 **Optical mapping video of** **Figure 4A** **.**
(WMV)

Movie S3 **Optical mapping video of** **Figure 4B** **.**
(WMV)

Movie S4 **Optical mapping video of** **Figure 4D** **.**
(WMV)

Movie S5 **Optical mapping video of** **Figure 5B** **.**
(WMV)
