# Supplementary material for: In Situ Optical Mapping of Voltage and Calcium in the Heart
Source: PLoS One. 2012 Aug 2;7(8):e42562. doi: 10.1371/journal.pone.0042562 (PMC3411684; doi:10.1371/journal.pone.0042562)
Supplement: Figure S1 — More Optical transients of Figure 1 . (DOC) [file pone.0042562.s001.doc]

**Supplemental Figure I**

**
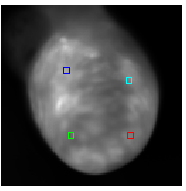
**

**Supplemental Figure I. Simultaneous imaging of Vm and [Ca^2+^]_i_ in a Langendorff-mode saline-perfused rat heart.** Four sets of V_m_ and [Ca^2+^]_i_ fluorescence signals (y-axis: camera signals on a 16-bit scale, x-axis: time in milliseconds) taken from 4x4-pixel square regions. Scale bar = 5 mm.
